# Temperature Variation under Continuous Light Restores Tomato Leaf Photosynthesis and Maintains the Diurnal Pattern in Stomatal Conductance

**DOI:** 10.3389/fpls.2017.01602

**Published:** 2017-09-20

**Authors:** Mohammad S. Haque, Alexandra de Sousa, Cristiano Soares, Katrine H. Kjaer, Fernanda Fidalgo, Eva Rosenqvist, Carl-Otto Ottosen

**Affiliations:** ^1^Department of Crop Botany, Bangladesh Agricultural University Mymensingh, Bangladesh; ^2^Department of Food Science, Aarhus University Aarhus, Denmark; ^3^Departamento de Biologia, Biosystems and Integrative Sciences Institute, Faculdade de Ciências, Universidade do Porto Porto, Portugal; ^4^Department of Plant and Environmental Sciences, University of Copenhagen Copenhagen, Denmark

**Keywords:** tomato, gas exchange, leaf chlorosis, abscisic acid, reactive oxygen species, antioxidants

## Abstract

The response of tomato plants (*Solanum lycopersicum* L. cv. Aromata) to continuous light (CL) in relation to photosynthesis, abscisic acid (ABA) and reactive oxygen species (ROS) was investigated to improve the understanding of the development and/or alleviation of CL-induced leaf injury in constant and diurnal temperature fluctuations with similar daily light integral and daily mean temperature. The plants were grown in three photoperiodic treatments for 15 days; One treatment with a 16/8 h light/dark period and a light/dark temperature of 27/17°C (Control), two CL treatments with 24 h photoperiods, one with a constant temperature of 24°C (CLCT) and the other one with variable temperature of 27/17°C for 16/8 ho, respectively (CLVT). A diurnal pattern of stomatal conductance (*g_s_*) and [ABA] was observed in the plants grown in the control and CLVT conditions, while the plants in CLCT conditions experienced a significant decrease in stomatal conductance aligned with an increase in ABA. The net photosynthesis (*A*) was significantly reduced in CLCT, aligned with a significant decrease in the maximum rate of Rubisco carboxylation (*V*_cmax_), the maximum rate of electron transport (*J*_max_) and mesophyll diffusion conductance to CO_2_ (*g*_m_) in comparison to the control and CLVT. An increased production of H_2_O_2_ and O_2_^•-^ linked with increased activities of antioxidative enzymes was seen in both CL treatments, but despite of this, leaf injuries were only observed in the CLCT treatment. The results suggest that the diurnal temperature fluctuations alleviated the CL injury symptoms, probably because the diurnal cycles of cellular mechanisms were maintained. The ROS were shown not to be directly involved in CL-induced leaf injury, since both ROS production and scavenging was highest in CLVT without leaf chlorotic symptoms.

## Introduction

Continuous lighting (CL) has negative effects in many species including tomato (Hilman, 1956; Globig et al., 1997; Matsuda et al., 2014), potato (Stutte et al., 1996), and eggplant (Murage et al., 1997). These sensitive species exhibit multiple responses to CL such as leaf chlorosis, down-regulation of photosynthesis, accumulation of carbohydrates and acceleration of leaf senescence (Sysoeva et al., 2010; Velez-Ramirez et al., 2011). Current research highlighted some of the mechanisms behind CL-induced injury (Velez-Ramirez et al., 2014), but the exact causes of the injuries and their development are not known (Sysoeva et al., 2010). Excess carbohydrate accumulation, photooxidative pressure, continuous signaling to the photoreceptors and disruption of the circadian clock have been suggested to lead to CL-induced leaf injury (Velez-Ramirez et al., 2011). Recently, an unbalanced photosystem I (PSI) and photosystem II (PSII) excitation due to down regulation of the type III light harvesting chlorophyll *a*/*b* binding protein 13 (*CAB-13*) gene expression was shown to be responsible for injury development under CL in tomato leaves (Velez-Ramirez et al., 2014). Diurnal temperature fluctuation in CL conditions alleviated leaf chlorosis in tomato, but the mechanisms remain unclear (Hilman, 1956; Matsuda et al., 2014; Haque et al., 2015a).

In a previous study it was demonstrated that CL lowers the stomatal conductance (*g_s_*) almost linearly over time (Haque et al., 2015b), while a strong but brief 2 h temperature drop from 26 to 10°C during CL allowed tomato plants to retain a circadian rhythm of stomatal regulation (Ikkonen et al., 2015). Stomatal opening and closing in response to water-deficit conditions is regulated by the plant stress hormone abscisic acid (ABA) (Tallman, 2004; Daszkowska-Golec and Szarejko, 2013). Roses under CL did not show leaf chlorotic symptoms but a significantly higher *g_s_* indicated less responsive stomata under CL conditions compared to shorter photoperiod (Mortensen and Gilserød, 1999; Fanourakis et al., 2015). The CL resulted in stomata that were less sensitive to closing stimuli and the impaired ability of the stomata to respond to closing stimuli was related to the depletion of foliar ABA (Arve et al., 2013; Fanourakis et al., 2013a,b). However, the diurnal pattern of ABA with or without temperature variation under CL has not so far been investigated.

Several studies have reported that CL downregulates photosynthesis in tomato (Globig et al., 1997; Demers et al., 1998; Matsuda et al., 2014; Velez-Ramirez et al., 2014) and increase leaf carbohydrate contents (Bradley and Janes, 1985; Demers and Gosselin, 2002). Furthermore, CL decreased the maximum photochemical efficiency (F_v_/F_m_) and quantum yield (F′_q_/F′_m_) of PSII in tomato plants grown in CL compared to plants grown in a 16 or 12 h photoperiod (Matsuda et al., 2014; Velez-Ramirez et al., 2014; Haque et al., 2015a). An increase in soluble sugars and starch in CL-grown tomato leaves may overload the Calvin–Benson cycle and limit CO_2_ fixation rate (Demers and Gosselin, 2002), but this has not been confirmed (Haque et al., 2015a). Also the effects of CL on maximum Rubisco carboxylation rate (*V*_cma_*_x_*) and maximum light and CO_2_ saturated electron transport (*J*_max_) are contradictory. CL grown cucumber plants experienced an increase in these parameters compared to 20 h light grown plants (Pettersen et al., 2010) whereas, CL decreased *V*_cmax_ and *J*_max_ in onion and in cultivated tomato species in comparison to plants grown in shorter photoperiods (Gestel et al., 2005; Velez-Ramirez et al., 2014).

Unbalanced photosynthesis under CL conditions may induce photooxidative damages by the generation of harmful reactive oxygen species (ROS) such as singlet oxygen (^1^O_2_), superoxide radial (O_2_^•-^), hydroxyl radical (OH^•^) and hydrogen peroxide (H_2_O_2_) (Murage and Masuda, 1997; Velez-Ramirez et al., 2011). In line with this, increased contents of enzymatic and non-enzymatic scavengers of ROS have also been found in CL-grown plants (Murage and Masuda, 1997; Demers and Gosselin, 2002), suggesting that plants grown under CL balances the damaging behavior of ROS by increased ROS scavenging (Gill and Tuteja, 2010).

In the present study, we studied the response of tomato plants to CL under constant and diurnal temperature fluctuations to elucidate the role of stomatal conductance, ABA, photosynthesis, photorespiration and the ROS production/scavenging balance on the ability of tomato plants to withstand CL-induced leaf injury. We hypothesized that diurnal temperature fluctuations would alleviate damages by restoring photosynthesis, entraining diurnal stomatal conductance and [ABA] and by increasing photorespiration. To clarify the relationship between photooxidative damage and leaf injury under CL, the formation of ROS and their scavenging activities were investigated in detail.

## Materials and Methods

### Plant Materials and Growth Conditions

Seeds of tomato (*Solanum lycopersicum* L. cv. Aromata) were sown in June 2014 in trays (60 pots of 40 mm diameter, Ellegaard A/S, Esbjerg, Denmark) containing Kekkila Ellepress mix (Kekkila Oy, Vantaa, Finland). The Kekkila express mix contained sphagnum peat with 1.0 kg m^-3^ N-P_2_O_5_-K_2_O and 10 kg m^-3^ dolomite limestone. The trays were kept in a climate controlled growth room where seed germination and seedling establishment took place under a 16 h photoperiod with a Photosynthetic Photon Flux Density (PPFD) of 250 μmol m^-2^ s^-1^, 25°C light/dark temperature, a relative humidity (RH) of 40–50% and 400 μmol mol^-1^ CO_2_. The supplemental light was provided by; Green power LED production module deep red/white 150 lamps (35W, Philips, Einhoven, The Netherlands). Two weeks after sowing, seedlings were transferred to 14 cm diameter plastic pots containing commercial peat potting mix (Pindstrup 2, Pindstrup Mosebrug A/S, Ryomgaard, Denmark). The seedlings were moved from the growth room and randomly allocated to three identical climate chambers (9.68 m^3^, Mb Teknik, Brøndby, Denmark) for 10 days in a 16 h photoperiod to acclimate the plants into a new light/dark cycle [light period 23:01–15:00 h and dark period 15:01 to 23:00 Central European Standard Time (CEST)]. This was done to ease data recording and handling of plants. The climate conditions were 350 μmol m^-2^ s^-1^ PPFD, 27/17°C light/dark temperature, 400 μl l^-1^ CO_2_ and 70/50% light/dark RH to keep a stable vapour pressure deficit (VPD) of 1.05 kPa. The light was provided by FL300 Sunlight LED fixtures (Senmatic A/S, Søndersø, Denmark).

### Light Treatments

After acclimation, the 4 weeks old plants were exposed to three photoperiodic treatments as outlined in **Table 1**; a 16 h photoperiod with light/dark temperature set points of 27/17°C (Control), a 24 h photoperiod with a constant 24°C temperature (CLCT) and a 24 h photoperiod where the temperature pattern was 16 h with 27°C and 8 h with 17°C (CLVT). The PPFD for control and CL treatments were approximately 350 and 230 μmol m^-2^ s^-1^, respectively to maintain identical daily light integral (DLI) of approximately 20 mol m^-2^ d^-1^ in the three treatments. The mean daily temperature in the three treatments was approximately 24°C. The light/dark period corresponding to the 16 h light and 8 h dark in the control treatment was similar to the two time periods in the CL treatments. The settings for RH were different in the three treatments to maintain a similar VPD of 1 kPa in all treatments: 70/50% RH in the light/dark periods in the control, constant 65% in CLCT and 70/50% for the 16 h 27°C/8 h 17°C period in CLVT. The similar VPD minimized interacting moisture stress differences between the alternating and constant temperature conditions in the three treatments. The CO_2_ concentrations inside the climate chambers were 400 μl l^-1^ and the light intensity was measured at the canopy level by a quantum sensor (LI-250A, LI-COR, Lincoln, NE, United States). The temperature, RH and CO_2_ concentration inside the climate chambers were recorded every 10 min by the climate computer. The plants were irrigated 1–3 times a day (depending on age) with a complete nutrient solution by an automatic timer controlled ebb/flood system.

**Table 1:** Diurnal climate data applied for the three growth conditions (Control, CLCT, and CLVT).

Treatments	Control		CLCT		CLVT	
Time of day	2301–1500	1501–2300	2301–1500	1501–2300	2301–1500	1501–2300
Light/dark (L/D)	L	D	L	L	L	L
PPFD (μmol m^-2^ s^-1^)	350	0	230	230	230	230
Temperature (°C)	27	17	24	24	27	17
RH (%)	70	50	65	65	70	50
VPD (kPa)	1	1	1	1	1	1

*Vapour pressure deficit (VPD) is calculated on the basis of given temperature and RH.*

### Photosynthetic Measurements

At the start of the experiment, the tomato plants had developed five leaves, where the youngest leaf was not fully unfolded (10–15 cm in length). This leaf was tagged to track the direct effect of the photoperiodic treatments on the following 5–7 leaves. Prevailing the physiological measurements during the experiment were done on the 1st and 2nd lateral leaflets of the 7th leaf, though stomatal conductance (*g_s_*) was measured on the 5th leaf on day 4 as the 7th leaf had not unfolded. The *g_s_* was measured using a porometer (PMR-2, PP systems, PP systems, Amesbury, MA, United States) at 8:00 CEST (9 h after the onset of the light period) and at 18:00 CEST (3 h after the onset of the dark period) on days 4, 8, 11, and 15. As there were no dark periods for CL measurements, the dark samples data of CL treatments meant the data those were taken at the same time point during the dark period of control. Photosynthetic leaf gas exchange (Net assimilation of CO_2_, *A* and *g_s_*) was measured using a portable gas analyser (CIRAS-2, PP systems, Amesbury, MA, United States) at 10 h after the onset of the light period (starting at 9:00 CEST) on day 14 of the CL treatment. The measurements were carried out under identical climate conditions for all treatments (PPFD 350 μmol m^-2^ s^-1^, temperature 27°C and 400 μl l^-1^ CO_2_). Furthermore, *A/C*_i_ curves were made for three plants from each treatment on day 13 (8.5 h after the onset of the light period, starting at 07:30 CEST) using the protocol by Ainsworth et al. (2002). The *A* was measured at 12–13 CO_2_ levels at a leaf temperature of 24°C and a saturated PPFD of 1200 μmol m^-2^ s^-1^. The saturated PPFD and optimum temperature for *A* were obtained from earlier photosynthetic light and temperature response curve for this cultivar. The *V*_cmax_, *J*_max_, *g*_m_ and photorespiration were estimated from the *A/C*_i_ curves using the formula from Ethier and Livingston (2004). The maximum photochemical efficiency [F_v_/F_m_ = (F_m_-F_o_)/F_m_] and the quantum yield [F′_q_/F′_m_ = (F′_m_-F_s_)/F′_m_] of PSII were measured by a MINI-PAM (Walz, Effeltrich, Germany) on day 12 at 08:00 CEST (9 h after the onset of the light period). The chlorophyll fluorescence data were measured with the same method as described by Haque et al. (2014). Relative Rubisco content was performed using the method of Soares et al. (2016). For each sample, gel slices corresponding to the large and small subunits of Rubisco were incubated 12 h in 2 mL of formamide at 50°C. At the same time, the remaining gel was incubated in the same conditions. The absorbance of the wash solutions was measured at 595 nm and the relative Rubisco content was calculated considering the values of absorbance at 595 nm. Results were expressed in percentage as Abs595RC/Abs595PC, in which RC is the Rubisco content and PC is the total protein content.

### Leaf Pigments and Carbohydrates

Samples for leaf chlorophyll content were collected at the end of light period on day 15, whereas the samples for leaf carbohydrate content were collected at the end of light period and end of dark period on day 8 and day 15. Six randomly selected leaflets from four plants from each treatment were collected and immediately plunged into liquid nitrogen and stored at -80°C until analysis. Before extraction, the leaf samples were freeze dried and ground in a mixer mill (MM200, Retsch, Inc., Haan, Germany) with a stainless steel ball (diameter 7 mm). The soluble sugars and pigments were extracted with 80% ethanol and 5 mM HEPES solution until the leaf material went pale. The leaf pigments in the supernatant were analyzed by light spectroscopy using a UV-VIS spectrophotometer (Shimadzu, Kyoto, Japan). Chlorophyll (Chl) *a* and Chl *b* concentrations were calculated from the measurements at three absorbance levels: 470, 648.6, and 664.2 nm (Lichtenthaler, 1987). The soluble sugars were analyzed by ion chromatography using a pulsed amperometic detector (PAD) with a gold electrode (Dionex, ICS 3000, Sunnyvale, CA, United States) using 200 mM NaOH as eluent. The ion chromatograph was equipped with a Dionex CarboPac PA1 carbohydrate column. The pellet of pale leaf material containing starch was incubated for 90 min in an autoclave at 120°C to gelatinise the starch. Thereafter the starch was broken down to glucose units with Na-acetate buffer containing amyloglucosidase and α-amylase at 30°C for 16 h. The samples were centrifuged for 5 min at 13414 *g*, and the supernatant was collected and filtered through a 0.45 μm syringe filter before analysis. The concentration of glucose units from starch were determined by ion chromatography equivalent to the method described above.

### Absiscic Acid (ABA) Determination

Two to three leaflets from the 7th leaf (from six plants) were collected at 07:00 and 19:00 CEST on day 8 and day 15 and immediately plunged into liquid nitrogen and stored at -80°C until analysis. The freeze-dried samples were ground and homogenized with a Genogrinder 2010 (Spex SamplePrep, Metuchen, NJ, United States). The extraction was carried out by the accelerated solvent extraction (ASE) method (Pedersen et al., 2011) using an automated extraction system (Dionex ASE 350, Hvidovre, Denmark). ABA was quantified using the commercial standard of ABA (Sigma-Aldrich, Brøndby, Denmark) and HPLC grade solvents and MilliQ water was used for extraction and analysis. The chromatographic separation was performed using a HPLC system (Agilent 1100, Single Quad, Agilent, Hørsholm, Denmark) equipped with a diode array detector The compounds were analyzed and estimated by negative mode ionization combined with API-ES mode and reversed-phase HPLC. The chromatographic analysis was carried out at a flow rate of 0.2 ml min^-1^ with an injection volume of 50 μL. A synergy 4 μ POLAR-RP 80A column of 250 mm × 2.0 mm size (Phenomenex, Cheshire, United Kingdom) with a column temperature of 30°C was used. The mobile phase consisted of 7% acetonitrile with 20 mM acetic acid (solvent A) and 78% acetonitrile with 20 mM acetic acid (solvent B) for chromatographic separation. A gradient profile with the following proportions of solvent B was applied [time (min), %B]: (0, 20), (2, 20), (4, 100), (6, 100), (6.5, 20), and (19, 20). All the compounds were detected at a wavelength of 254 nm. The chemical compounds were identified by comparing the fragmentation pattern and relative retention times with their pure reference compounds. The data were analyzed using the software Agilent Chemstation (version B.04.03).

### Determination of Reactive Oxygen Species (ROS) and Antioxidative Enzymes

Two to three leaflets from the 7th leaf (for each sample) were collected at 07:00 and 19:00 CEST (8 and 4 h after the onset of the light and dark periods, respectively) on day 8 and day 15 and immediately plunged into liquid nitrogen and stored at -80°C until analysis. The level of superoxide anion was assayed as described by Doke (1983) with a minor modification of using frozen leaf samples instead of fresh leaf samples. The H_2_O_2_ quantification was performed according to de Sousa et al. (2013), with slight modifications. H_2_O_2_ was extracted from leaves (0.5 g) frozen in liquid nitrogen and macerated in mortars at 4°C with 1.2 mL of potassium phosphate buffer (50 mM, pH 6.5). The homogenate was centrifuged at 6000 *g* for 25 min at 4°C. To determine H_2_O_2_ levels, 1 mL of extracted solution was mixed with 1 mL of 0.1% (w/v) titanium sulfate in 20% (v/v) H_2_SO_4_, and the mixture was then centrifuged at 6000 *g* for 15 min at 4°C. The absorbance of the mixture was measured at 410 nm and H_2_O_2_ levels were calculated using the extinction coefficient 0.28 μM^-1^ cm^-1^. The SOD (EC 1.15.1.1) activity was assayed by the inhibition of the photochemical reduction of nitroblue tetrazolium (NBT) as described by Donahue et al. (1997). The CAT (EC 1.11.1.6) and APX (EC 1.11.1.11) activities were determined according to Amako et al. (1994) and Rao et al. (1996), respectively. Total antioxidant activity (TAC) was extracted in 1.5 mL of ice-cold 80% (v/v) methanol. Methanolic extracts (0.1 mL) were mixed with 1 mL of the *Reagent Solution* (0.6 M sulfuric acid, 28 mM sodium phosphate and 4 mM ammonium molybdate) and incubated at 95°C for 90 min. After reaching room temperature (25°C), samples were measured at 695 nm. Ascorbic acid was prepared in methanol 95% (v/v) and used as reference standard. Polyphenols were extracted in 5 mL of a mixture of 1% (v/v) methanol in 1% (v/v) HCL. Total phenolic content was determined using a Folin–Ciocalteu assay according to Singleton and Rossi (1965). Gallic acid was used as standard. Flavonoid content was measured using the modified aluminum chloride method (Chang et al., 2002), using quercetin as a standard.

### Statistical Analysis

The open source statistical software R (R Core Team, 2013) was used to evaluate the variation of parameters between the treatments. Data analysis was done using one-way analysis of variance (ANOVA) with a significance level of *P* < 0.05. The multiple comparisons of treatment means for each day of measurement was done considering the factor treatment by Tukey test and Waller–Duncan k ratio *t*-test.

## Results

**FIGURE 1:**
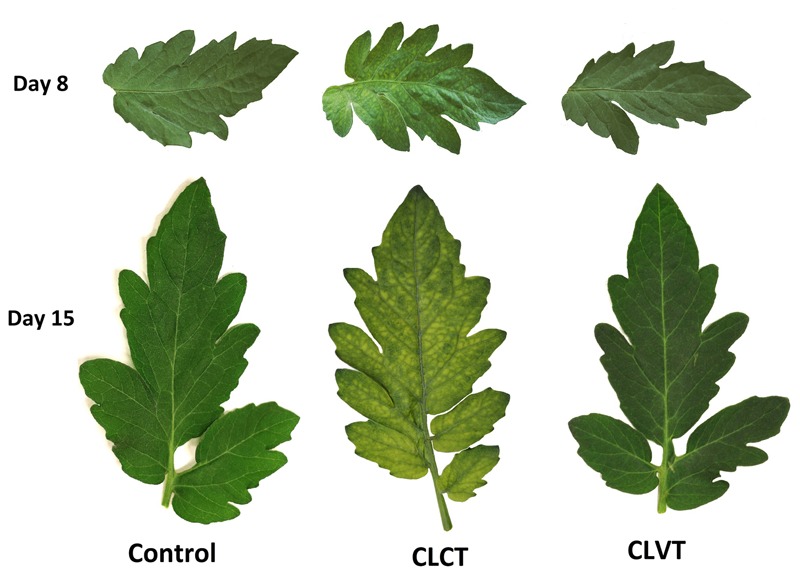
The leaflets of the 7th leaf from tomato plants grown in control conditions with a 16 h photoperiod, in continuous light with constant temperature (CLCT) and in continuous light with variable temperature (CLVT). The photographs were taken after 8 days (top row) and 15 days (bottom row) in the three treatments.

Leaf chlorosis was visible after 7 days of CL treatment on the 6th leaf and younger leaves in CLCT. The mottling initiated at the base of the leaflets and progressed toward the apex. All developing leaves starting from the 6th leaf to the top were chlorotic after 15 days. **Figure 1** shows leaflets from plants in the respective treatments where mild and severe chlorotic leaflets were present on day 8 and day 15 in the CLCT treatment.

### Stomatal Conductance and ABA

**FIGURE 2:**
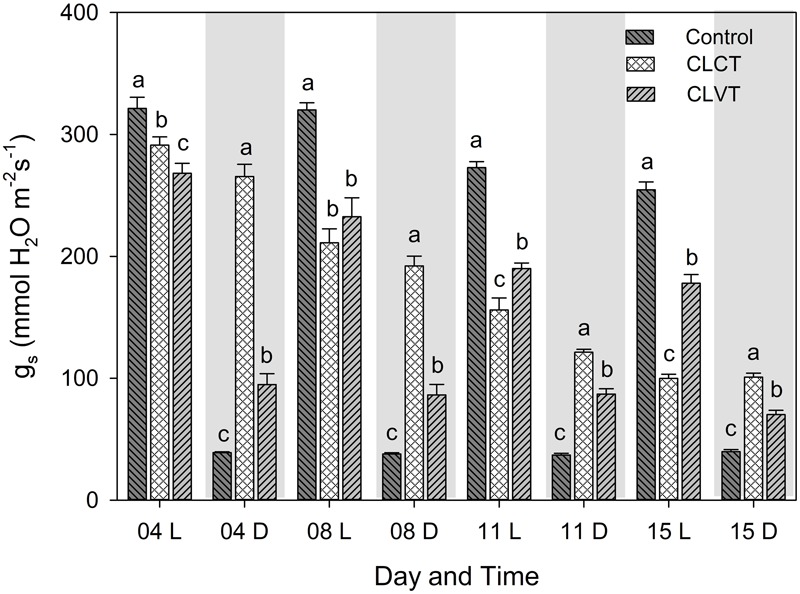
The diurnal pattern of stomatal conductance (*g_s_*) in the plants grown in control, CLCT and CLVT conditions. The measurements were taken at two time points during the respective days, the white and gray graph areas represent the light (L, 9 h after the onset of the light period, at 08:00 CEST) and dark period (D, 3 h after the onset of the dark period, at 18:00 CEST) under control conditions. Though, there were no dark periods for CL measurements, data were taken at the same time point as in the control. Vertical bars are SEM (*n* = 6). Treatment means with different letters within each time of the day are significantly different.

The *g_s_* decreased during the experiment period in both CL-treatments when compared with the control plants (**Figure 2**). For the CLVT-grown plants, the stomata closed partially in the low-temperature period and the diurnal pattern of *g_s_* was similar to the pattern seen in the control plants. In contrast, the decrease in *g_s_* was close to linear during the 15 days of CLCT and there was no diurnal pattern (**Figure 2**).

**FIGURE 3:**
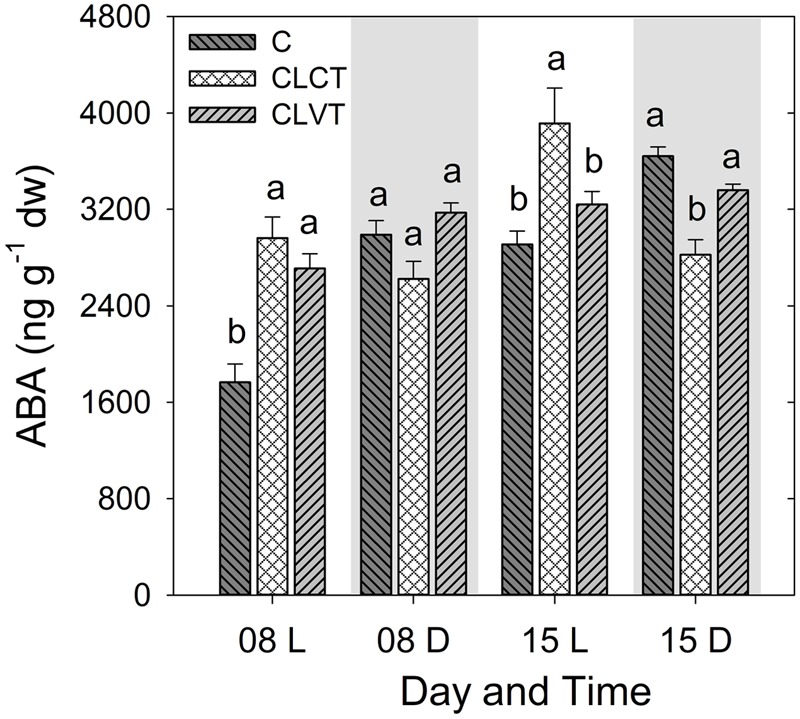
The abscisic acid (ABA) contents in leaves of tomato plants grown in control, CLCT and CLVT conditions. The samples were taken 8 h after the onset of the light period (L) at 7:00 CEST and 4 h after the onset of the dark period (D) at 19:00 CEST on days 8 and 15. Vertical bars are SEM (*n* = 6). Treatment means with different letters within each day are significantly different.

For leaf [ABA], the diurnal pattern with higher values in the dark period and lower values in the day period seen in the control plants was not seen for plants grown in CL conditions. Leaf [ABA] was significantly lower in the light period of the control plants compared to plants grown in CL on day 8, though this pattern was only seen in relation to the CLCT-grown plants on day 15 (**Figure 3**). No significant differences were seen among treatments during the period corresponding to the dark period on day 8, whereas leaf [ABA] was significantly lower in the CLCT-grown plants compared to the other treatments on day 15 (**Figure 3**).

### Chlorophyll Fluorescence and Pigments

The F_v_/F_m_ and F′_q_/F′_m_ values were significantly lower in the leaves of CLCT-grown plants compared to plants grown in the two other treatments on day 12 (**Table 2**), whereas no significant differences were seen between the control and the CLVT treatment. Despite the same F′_q_/F′_m_, the CLVT grown plants had lower electron transport rate (ETR) than plants grown in the control due to the lower *in situ* PPFD in the CL conditions. The leaf Chl *a* + *b* content was significantly reduced in plants grown in CLCT, followed by CLVT and control plants on day 15 (**Table 2**). A significantly increased level of relative Rubisco content was found in the CLVT plants compared to the CLCT- and control plants during the light period of day 15 (**Table 2**).

**Table 2:** The Chl *a* + *b* (mg g^-1^ DW), maximum photochemical efficiency of PSII (F_v_/F_m_), quantum yield of photosystem II (F′_q_/F′_m_) and electron transport rate (ETR) in the leaves of plants grown in control, CLCT and CLVT conditions.

Parameters	Treatments		
	Control	CLCT	CLVT
Chl *a* + *b* (mg g^-1^ dw)	19.2 ± 0.3 a	13.5 ± 0.5 c	17.1 ± 0.3 b
F_v_/F_m_	0.81 ± 0.003 a	0.60 ± 0.025 b	0.80 ± 0.006 a
F′_q_/F′_m_	0.679 ± 0.004 a	0.471 ± 0.034 b	0.672 ± 0.002 a
ETR	95 ± 0.5 a	45 ± 3.3 c	65 ± 0.2 b

*The leaf Chl content was measured at day 15 and F_*v*_/F_*m*_ and F′_*q*_/F′_*m*_ values were recorded at day 12 of CL exposure. The F’_*q*_/F’_*m*_ was measured at the *in situ* PPFD of 350 μmol m^-*2*^ s^-*1*^ for the control plants and 230 μmol m^-*2*^ s^-*1*^ for the CL-grown plants, for CLVT during the high temperature period. Vertical bars are SEM (*n* = 6). Treatment means with different letters within each day are significantly different.*

### Photosynthesis

**FIGURE 4:**
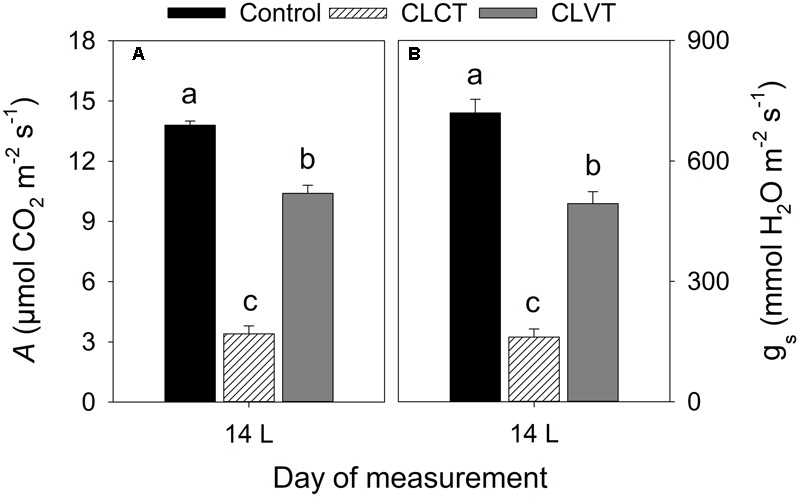
**(A)** The rate of leaf photosynthesis (*A*) and **(B)** *g_s_* measured at a PPFD of 350 μmol m^-2^ s^-1^, 27°C and a CO_2_ level of 400 μmol mol^-1^ on day 14 for plants of all treatments. Vertical bars represent SEM (*n* = 6). Treatment means with different letters within each day are significantly different.

**FIGURE 5:**
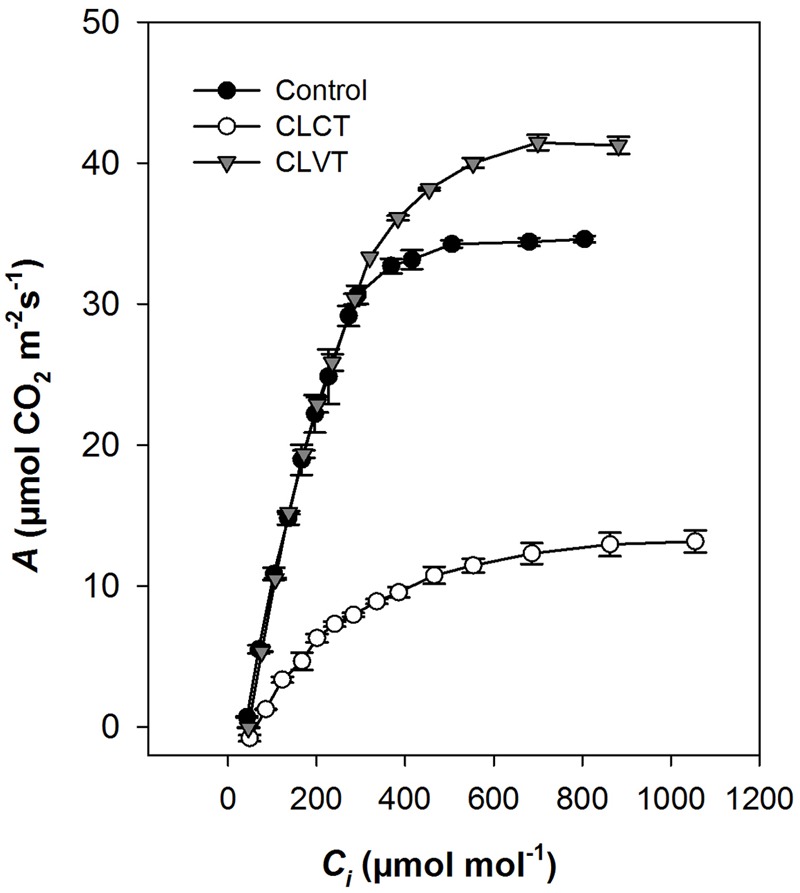
Response of photosynthesis to intercellular CO_2_ (*A*/*C_i_*) in plants grown in control, CLCT and CLVT conditions. Measurements were made at saturating PPFD (1200 μmol m^-2^ s^-1^) and 24°C temperature on day 13 of CL exposure. Vertical bars are SEM (*n* = 3). Lines were drawn by fitting the predicted parameters (**Table 3**) described by Ethier and Livingston (2004).

At a PPFD of 350 μmol m^-2^ s^-1^ and at 400 ul l^-1^ CO_2_, a significant reduction in *A* and *g_s_* were observed in the chlorotic leaves of the CLCT grown plants in comparison to the values measured on non-chlorotic leaves in the control and CLVT grown plants (**Figures 4A,B**). At a saturated PPFD of 1200 μmol m^-2^ s^-1^ the *A*/*C*_i_ response curves showed that *A* was limited already at 400 ul l^-1^ CO_2_ in the CLCT treatment (**Figure 5**), and the calculated values of *V*_cmax_, *J*_max_, *g*_m_ and photorespiration were significantly lower in CLCT followed by the CLVT and control plants (**Table 3**). Furthermore, the *J*_max_/*V*_cmax_ ratio was significantly lower in the CLCT grown plants than in the CLVT- and control plants, and in contrast a significantly higher *C*_i_/*C*_a_ measured at 400 μmol mol^-1^ *C*_a_ was observed for the CLCT plants in comparison to CLVT and control plants (**Table 3**).

**Table 3:** The maximum rate of Rubisco carboxylation (*V_cmax_*), maximum rate of electron transport (*J_max_*), *J_max_/V_cmax_* ratio, mesophyll conductance diffusion to CO_2_ (*g_m_*), *C_i_*/*C_a_* at 400 μmol mol^-1^ *C_a_*, photorespiration obtained from *A/C_i_* curve **(**Figure 5**)** at day 13 and relative Rubisco content at day 15 of CL exposure in the leaves of control, CLCT and CLVT grown plants.

Parameters	Treatments		
	Control	CLCT	CLVT
*V_cmax_* (μmol CO_2_ m^-2^s^-1^)	80 ± 1 b	51 ± 1 c	101 ± 3 a
*J_max_* (μmol e^-^ m^-2^s^-1^)	174 ± 0.5 b	67 ± 2 c	204 ± 3 a
*J_max_/V_cmax_* ratio	2.18 ± 0.02 a	1.32 ± 0.02 b	2.02 ± 0.04 a
*g_m_* (mol CO_2_ m^-2^s^-1^)	0.15 ± 0.01 ab	0.10 ± 0.02 b	0.18 ± 0.01 a
*C_i_*/*C_a_* at 400 μmol mol^-1^ *C_a_*	0.68 ± 0.001 b	0.84 ± 0.01 a	0.72 ± 0.01 b
Photorespiration (μmol CO_2_ m^-2^s^-1^)	8.5 ± 0.03 b	3.8 ± 0.49 c	10.5 ± 0.28 a
Relative Rubisco content (%)	22 ± 1.8 b	26 ± 0.5 b	32 ± 1.5 a

*Vertical bars are SEM (*n* = 3). Treatment means with different letters within each day are significantly different.*

### Carbohydrates

**FIGURE 6:**
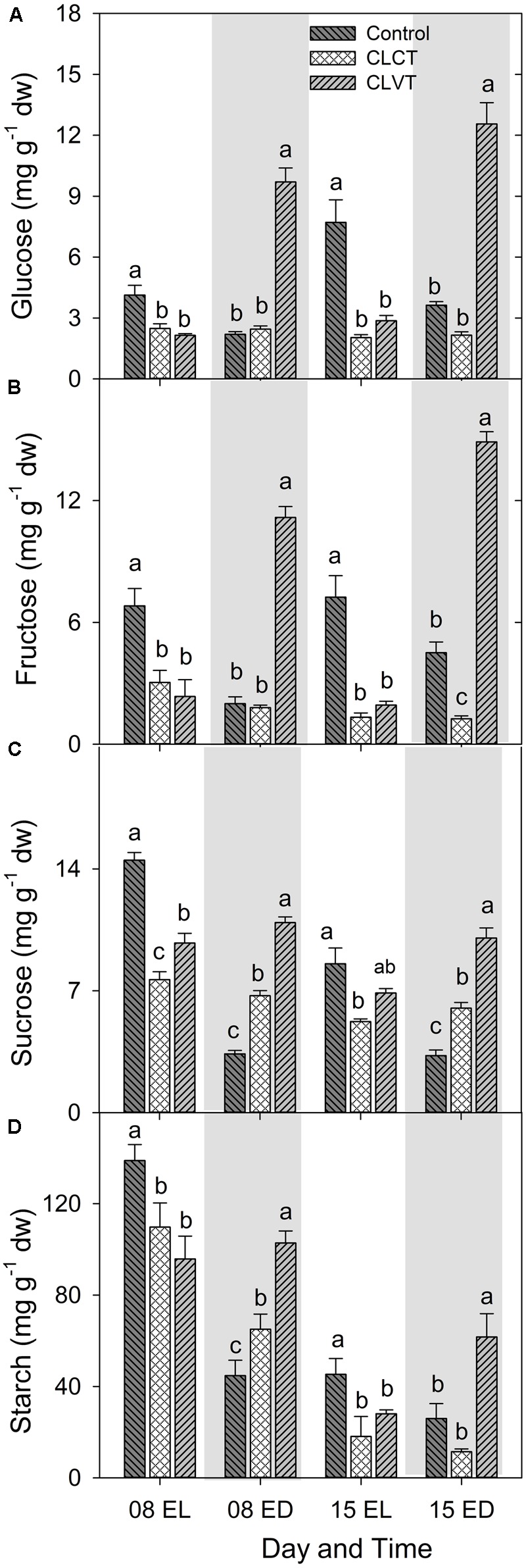
Glucose **(A)**, fructose **(B)**, sucrose **(C)**, and starch **(D)** contents in leaves of plants grown in control, CLCT and CLVT conditions. The samples were collected at the end of light period (EL, 15:00 CEST) and at the end of dark period (ED, 23:00 CEST) on days 8 and 15. Vertical bars are SEM (*n* = 6). Treatment means with different letters within each day are significantly different.

A diurnal pattern in leaf carbohydrate contents resulted in high values of hexoses (glucose and fructose), sucrose and starch by the end of the light period (EL), and low values of carbohydrates by the end of the dark period in the control plants (**Figure 6**). However, this pattern was more or less opposite for the CLVT-grown plants, whereas no diurnal pattern was present in the CLCT-grown plants. The leaf starch content decreased significantly from day 8 to day 15 in all three treatments (**Figure 6D**).

### ROS and Antioxidants

**FIGURE 7:**
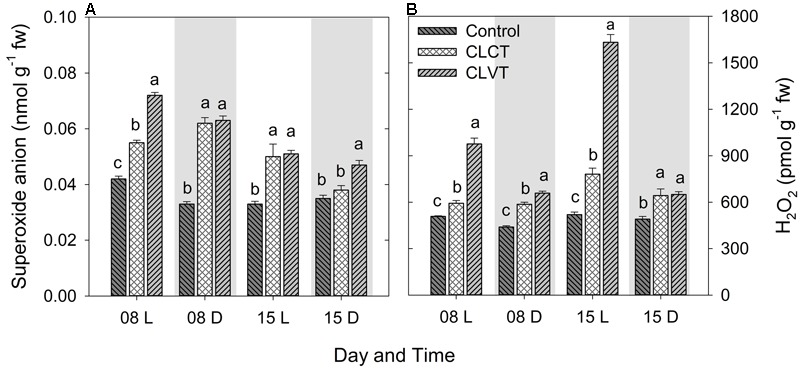
The superoxide anion **(A)** and hydrogen peroxide **(B)** contents in the leaves of tomato plants grown in control, CLCT and CLVT conditions during the light (L) and dark (D) period of days 8 and 15 of CL exposure. The legends are same as in **Figure 3**. Vertical bars represent SEM (*n* = 4). Treatment means with different letters within each day are significantly different.

Continuous light significantly increased both the superoxide anion (O_2_^•-^) and the H_2_O_2_ concentrations in the leaves compared to the control plants (**Figure 7**). The O_2_^•-^ level was significantly higher in the leaves of CLVT plants compared to CLCT plant in the light period of day 8 and in the dark period of day 15. The H_2_O_2_ content increased to higher levels during the light period when the CL conditions were combined with temperature fluctuations in comparison to constant temperatures (**Figure 7B**).

**FIGURE 8:**
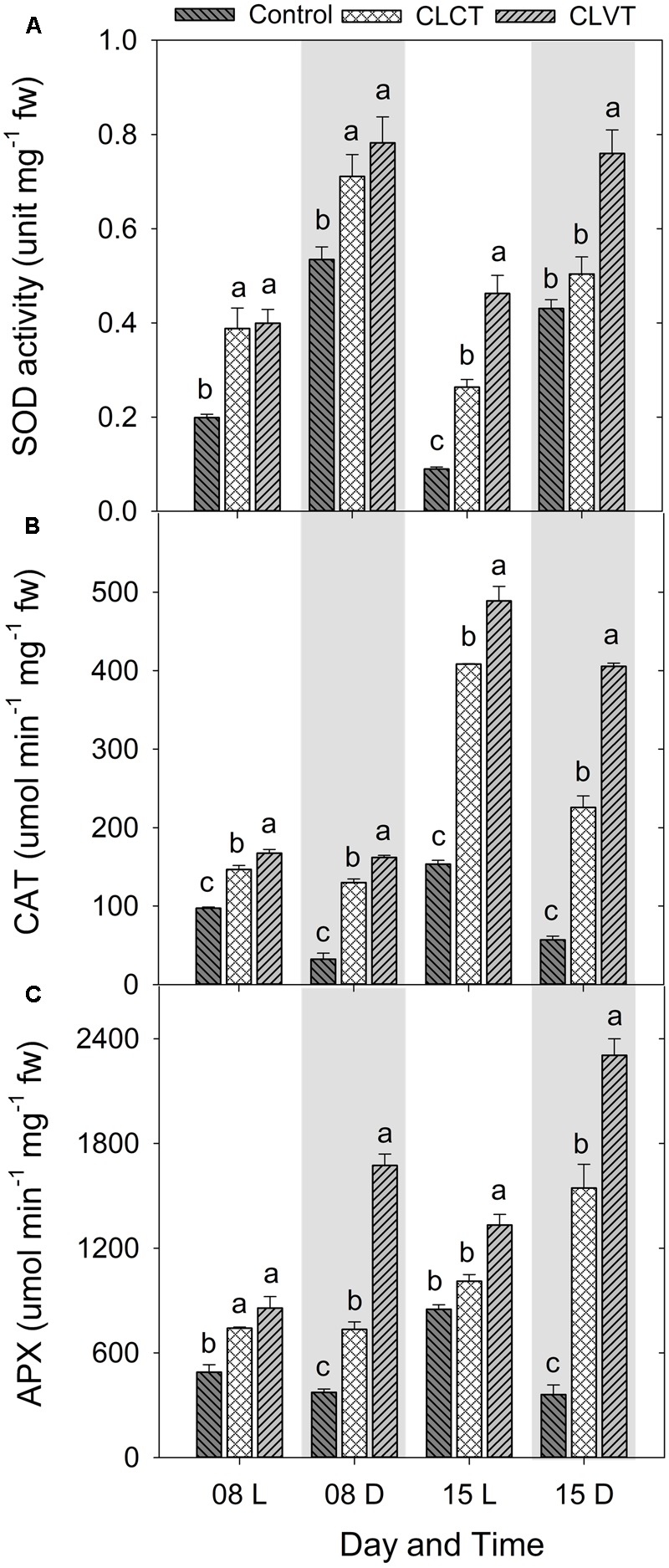
**(A)** Superoxide dismutase (SOD), **(B)** catalase (CAT), and **(C)** ascorbate peroxidase (APX) enzyme activities in the leaves of tomato plants grown in control, CLCT and CLVT conditions during the light (L) and dark (D) period of days 8 and 15 of CL exposure. The legends are same as in **Figure 3**. Vertical bars represent SEM (*n* = 3). Treatment means with different letters within each day are significantly different.

**FIGURE 9:**
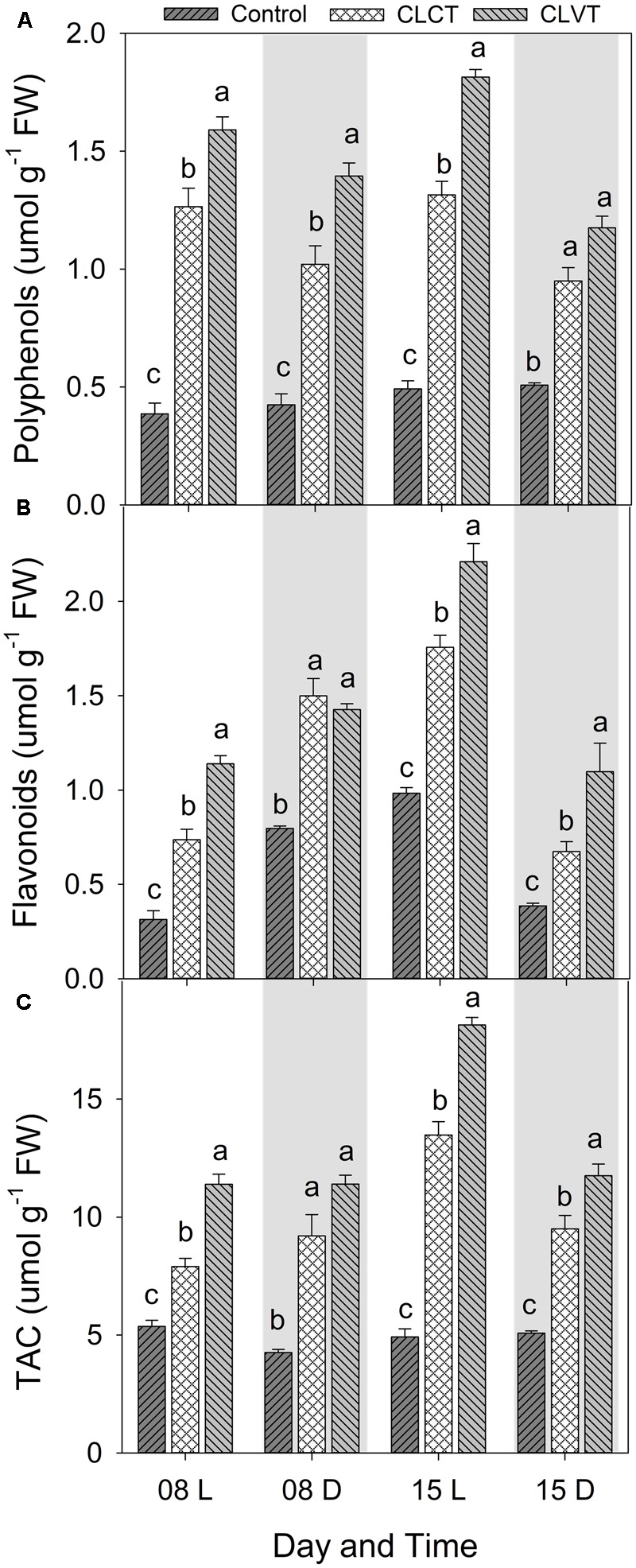
Polyphenols **(A)**, Flavonoids **(B)**, and total antioxidant activity (TAC, **C**) in the leaves of tomato plants grown in control, CLCT and CLVT conditions during the light (L) and dark (D) period of days 8 and 15 of CL exposure. The legends are same as in **Figure 3**. Vertical bars represent SEM (*n* = 3). Treatment means with different letters within each day are significantly different.

**FIGURE 10:**
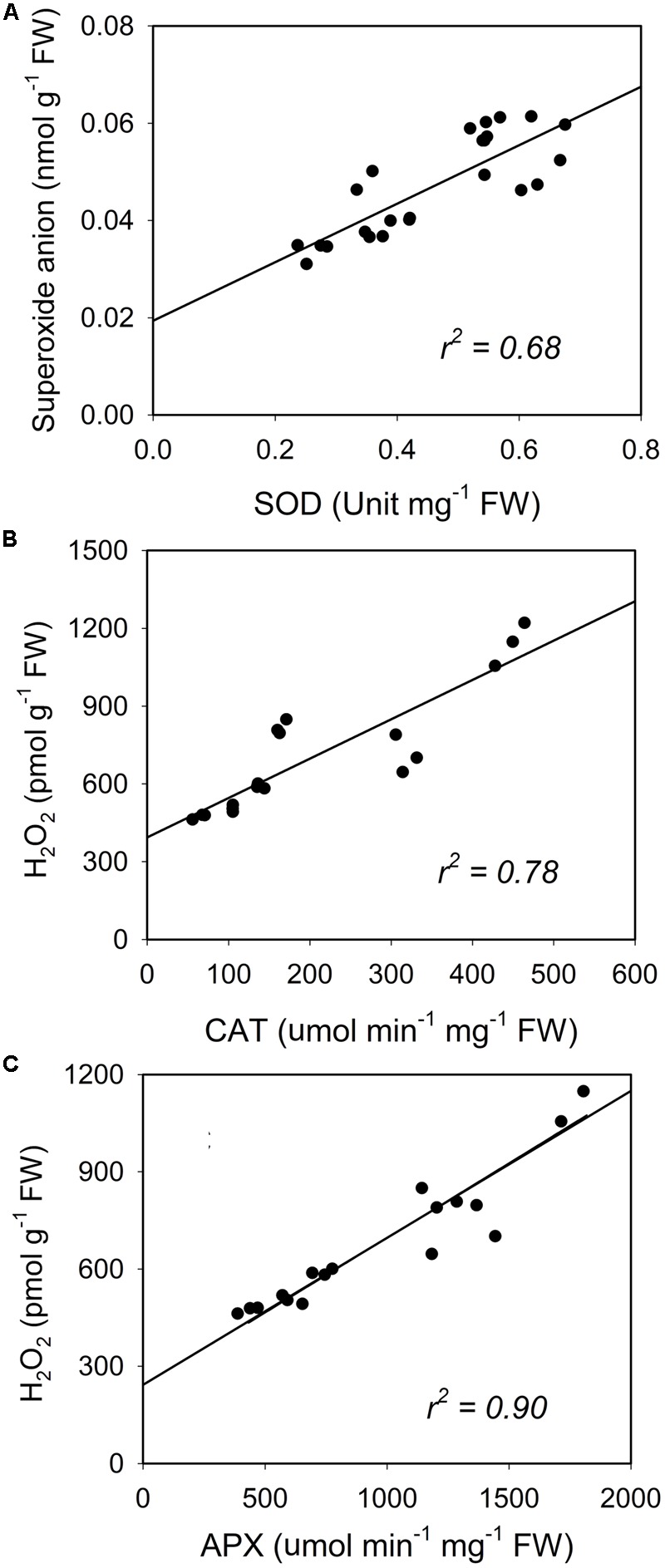
Reactive oxygen species (ROS) (superoxide anion and H_2_O_2_) plotted in relation to the superoxide dismutase (SOD, **A**) and the activities of catalase (CAT, **B**) and ascorbate peroxidase (APX, **C**). Each dot represent an average value of light and dark values for each day in each treatment.

A similar increase was seen in SOD and the enzymatic activities of CAT and APX (**Figure 8**). Activity of the antioxidative enzymes generally increased significantly in the CL-grown plants compared to control plants (**Figure 8**). However, the CLVT-grown plants experienced a further increase in SOD and CAT activity on day 15. **Figures 8A,B**, whereas APX activity was higher at any time of measurement in CLVT compared to the CLCT-grown plants (**Figure 8C**). An increasing trend of CAT and APX enzyme activities in the leaves of CL were observed from day 8 to day 15 (**Figures 8B,C**). The non-enzymatic antioxidants such as polyphenols, flavonoids and TACs also increased in the CL-grown plants in comparison to the control plants (**Figure 9**). These antioxidant activities were significantly higher in CLVT followed by CLCT and control plants especially during the light period (**Figures 9A–C**). A correlation analysis was carried out to illustrate the balance between daily average values of ROS production and ROS scavenging (**Figure 10**). We found near-linear correlations between O_2_^•-^ levels and SOD levels (**Figure 10A**), H_2_O_2_ and CAT levels (**Figure 10B**), and H_2_O_2_ and APX levels (**Figure 10C**) across the three treatments, showing that increased production of ROS induced high scavenging activity thereby reducing the damaging effects of the ROS.

## Discussion

### Continuous Light Reduces Photosynthesis Due to Damage to PSII and Low Rates of RuBP Regeneration

The significant decline in F_v_/F_m_ and F′_q_/F′_m_ in CLCT (the latter in comparison to CLVT with the same *in situ* PPFD) reflected that continuous light in combination with constant temperature causes serious damage to PSII. These damages were followed by degradation of chlorophyll and lower photosynthetic rates. Furthermore, the results showed that *A* was limited by both Rubisco carboxylation and RuBP regeneration. In contrast, a 10°C daily variation in temperature was sufficient to restore leaf photochemistry and maintain photosynthetic rates close to the values seen in the control plants. The lower *J*_max_/*V*_cmax_ ratio in the CLCT plants suggested that *A* was mainly limited by RuBP regeneration as shown by Yamori et al. (2011). The high rate of photorespiration seen in the treatments with diurnal temperature variation was caused by a higher rate of RuBP oxygenation resulting in enhanced RuBP regeneration and subsequently maintained the steady rate of photosynthesis despite of the continuous light. We further suggest that the lower ratio of *C*_i_/*C*_a_ due to high *A* caused a low CO_2_/O_2_ ratio, which may also have increased the rate of RuBP oxygenation over RuBP carboxylation.

Furthermore, the lower *g*_m_ in CLCT determines a limitation of *A* by a movement of CO_2_ from the suboptimal cavity (*C*_i_) to the site of carboxylation (chloroplast, *C*_c_). However, it has earlier been suggested that small reductions in *g*_m_ does not efficiently decrease the concentration of CO_2_ at the site of carboxylation to a degree where it can impose an effect on photosynthesis (Niinemets et al., 2009). Matsuda et al. (2014) found a significant decrease in photosynthesis in tomato plants grown in CL with constant temperature in comparison to plants grown in a 12 h photoperiod and hypothesized that *A* was limited by reduced RuBP carboxylation and not caused by reduced *g*_m_ due to stomatal closure. Our results confirm this hypothesis.

Recently, Velez-Ramirez et al. (2014) reported that *A* in cultivated tomato species was limited by a reduction in *V*_cmax_ and *J*_max_, which however, was not seen in a wild tomato species during CL. They suggested that the type III light harvesting chlorophyll a/b binding protein 13 (*CAB-13*) gene was responsible for the CL-tolerance in wild tomato species and that a downregulation of *CAB-13* in CL-grown cultivated tomato leaves led to an unbalanced PSI and PSII excitation resulting in leaf injury. The F_v_/F_m_ and *A* of CLVT-grown plants in our study performed similar to the wild species in Velez-Ramirez et al. (2014) and in our previous study (Haque et al., 2015a) resulting in no leaf injury. We suggest that the diurnal temperature fluctuations may somehow upregulate the expression of *CAB-13* or that a possible circadian clock regulated diurnal pattern in the expression of genes related to light-harvesting proteins are entrained by temperature.

### ROS Scavenging Balances ROS Production Averting Photooxidative Damage in Continuous Light

Earlier studies have suggested that photooxidative damage in plants grown in CL is due to increased activities of antioxidants (Murage and Masuda, 1997; Demers and Gosselin, 2002). Stepwise reduction of O_2_ by high energy exposure or electron transport reactions leads to the production of highly reactive ROS which damage the ultrastructure and function of chloroplasts, affecting PSII activity and photosynthetic pigments (Foyer et al., 1994). In our study, both CL treatments showed significantly higher production of H_2_O_2_ and O_2_^•-^ and especially the H_2_O_2_ formation was high in CLVT, and possibly related to an increased photorespiratory C recycling pathway enhancing the obligatory production of H_2_O_2_ in the peroxisomes through the glycolate oxidase reduction (Noctor et al., 2002). The antioxidative enzyme CAT predominantly scavenges H_2_O_2_ in the peroxisomes, while APX performs the same function in the cytosol and in the chloroplast (Das and Roychoudhury, 2014). APX has a higher affinity for H_2_O_2_ than CAT suggesting that APX is responsible for maintaining the low levels of H_2_O_2_ while CAT is responsible for the removal of its excess (Mittler, 2002). The increased activities of both CAT and APX in the CL treatments and the linear relationship with H_2_O_2_ show that the excess H_2_O_2_ production was scavenged averting cellular damage, especially in the CLVT plants. The increased SOD activities also played the same role and catalyzed the removal of O_2_^•-^ by dismutating it into O_2_ and H_2_O_2_. This was also confirmed by a linear relationship between O_2_^•-^ and SOD across the three treatments. The results suggest that there is a balance between ROS production and ROS scavenging in both CL treatments and that the development of leaf injury was not directly associated with photooxidative damage.

### Stomatal Regulation Linked Closely to the Circadian Clock Overrides the Effects of ABA Metabolites

It is well established that ABA controls stomatal opening and closing which is in turn regulated by the environmental factors of light and relative air humidity via VPD and temperature (Tallman, 2004). A dual-source model linking ABA-mediated regulation of diurnal stomatal movements proposed that darkness will favor guard cell biosynthesis of endogenous ABA and disfavor ABA catabolism (Tallman, 2004). In light, xanthophyll cycling, isomerisation of ABA precursors and activation of a cytochrome P450 mono-oxygenase (CytP450) would deplete endogenous guard cell ABA and liberate guard cells to extrude protons and accumulate the ions and water needed to increase guard cell turgor and open stomata (Tallman, 2004). Our results confirm a relationship between the diurnal pattern in stomatal opening and closure, and the diurnal pattern of lower [ABA] during the day and higher [ABA] during the night in the control plants. However, the two CL treatments did not show a diurnal pattern in [ABA] that could explain the opening and closure in the CLVT treatment or the linear closure of stomata in the CLCT treatment, although [ABA] daytime values were in general high in this treatment. Arve et al. (2013) showed that darkness is required for the biosynthesis of ABA from ABA-GE, which potentially would increase ABA and thereby stomatal closure in CL-grown plants, though this was not seen in our experiment. Instead, we suggest that the *in situ* leaf [ABA] does not reflect the sole source of diurnal stomatal movements or stomatal closing ability as suggested by Fanourakis et al. (2016). This assumption is further confirmed by recent results demonstrating that the ABA-GE/ABA ratio was a better predictor than ABA on the ability of stomata to close in basil grown in different light qualities (Jensen et al., 2017). Furthermore, the temperature variation in CLVT demonstrated an important role for the opening and closure of the stomata independently of the effects of [ABA]. A short temperature drop from 26 to 10°C for 2 h under CL conditions was recently also shown to reset the circadian rhythm of *g_s_* in tomato plants supporting the results in the current study (Ikkonen et al., 2015).

## Conclusion

The study shows clear diurnal patterns of stomatal conductance, ABA, carbohydrate metabolism and enzyme activities reflecting that plants respond to light by opening their stomata for increased photosynthesis and assimilation of carbohydrates. During the dark, increased ABA biosynthesis induced stomatal closure and the stored carbohydrates were degraded, and used for cellular maintenance. During the day, high activities of CAT and APX scavenged excess H_2_O_2_ from photorespiration, whereas higher SOD activity scavenged O_2_^-^ into H_2_O_2_. When the dark period was absent, stomata closed and carbohydrate levels became constant reflecting a continuous supply and utilization of photosynthates. The production of ROS increased due to increased photorespiration and a lethal effects of CL on the photosystems and on CO_2_ assimilation. Luckily, anti-oxidative enzymatic activity also increased leading to a steady-state balance in ROS production and scavenging to avoid photo-oxidative damages. If then a temperature variation was introduced to the constant light conditions, stomatal opening and closure immediately restored to follow the light:dark conditions of the control. Increasing the temperature opened the stomata and decreasing the temperature closed the stomata. This happened, irrespectively of a continuously high [ABA], and changes in the humidity introduced to counteract the temperature effects on VPD.

## Author Contributions

MH: contributed in planning and designing of the experiment, experimental set up, all data recording and sampling, biochemical analyses, statistical analysis and writing of the manuscript (major contribution). AdS and CS: performed the ROS, antioxidants and relative Rubisco content analyses; contributed to draft writing. KK: contributed in designing of the experiment, supervision and monitoring, biochemical analyses; critically revised the manuscript. FF: contributed in supervision of ROS, antioxidants and relative Rubisco content analyses. ER and C-OO: contributed in designing of the experiment, supervision and monitoring, manuscript writing.

## Conflict of Interest Statement

The authors declare that the research was conducted in the absence of any commercial or financial relationships that could be construed as a potential conflict of interest.
